# Supplementary material for: Learning to detect AI texts and learning the limits
Source: PLoS One. 2025 Oct 15;20(10):e0333007. doi: 10.1371/journal.pone.0333007 (PMC12527182; doi:10.1371/journal.pone.0333007)
Supplement: S2 File — All data and scripts used in the study, some additional descriptive statistics, scalable formate figures. (ZIP) [file pone.0333007.s002.tgz › data and scripts/OSF/scripts/analysis/mixed models jamovi/outputs/complete_correctness_learners.html]

Results


# Results

# Generalized Mixed Model

| Model Info | | | | | |
| --- | --- | --- | --- | --- | --- |
| Info | | Value | | Comment | |
| Model Type |  | Logistic |  | Model for binary y |  |
| Call |  | glm |  | Is correct ~ 1 + More readable is human + Confidence + Demographics: age + Demographics: education\_ordinal + Demographics: ai\_usage\_ordinal + Demographics: gender merged + Demographics: field\_merged + Reaction time log + GLS Euclidean distance + GLS1 difference + GLS2 difference + GLS3 difference + GLS4 difference + id + Demographics: reading\_disorder\_merged + GLS8 difference + GLS7 difference + GLS6 difference + GLS5 difference + Demographics: ai\_improve + Demographics: ai\_risk + Demographics: ai\_quality + Demographics: ai\_lacks\_creativity + Demographics: ai\_lacks\_understanding + Demographics: ai\_grammar + Demographics: ai\_avoidance + Demographics: ai\_dumbing + Demographics: ai\_fascination + (0 + More readable is human + Confidence + Reaction time log + GLS Euclidean distance + GLS1 difference + GLS2 difference + GLS3 difference + GLS4 difference + id + GLS8 difference + GLS7 difference + GLS6 difference + GLS5 difference \| Participant ID) |  |
| Link function |  | Logit |  | Log of the odd of y=1 over y=0 |  |
| Direction |  | P(y=1)/P(y=0) |  | P( Is correct = 1 ) / P( Is correct = 0 ) |  |
| Distribution |  | Binomial |  | Dichotomous event distribution of y |  |
| LogLikel. |  | -1173.143 |  | Unconditional Log-Likelihood |  |
| -2\*LogLikel. |  | 2346.287 |  | Unconditional absolute deviance |  |
| Deviance |  | 1705.411 |  | Conditional relative deviance |  |
| R-squared |  | NaN |  | Marginal |  |
| R-squared |  | NaN |  | Conditional |  |
| AIC |  | 2620.290 |  | Less is better |  |
| BIC |  | 3392.588 |  | Less is better |  |
| Residual DF |  | 1937.000 |  |  |  |
| Chi-squared/DF |  | 0.675 |  | Overdispersion indicator |  |
| Converged |  | yes |  |  |  |
| Optimizer |  | bobyqa |  |  |  |
|  |  |  |  |  |  |
| --- | --- | --- | --- | --- | --- |
| Note. R-squared cannot be computed. | | | | | |
| Note. boundary (singular) fit: see ?isSingular | | | | | |
|  | | | | | |
| [3] | | | | | |

## Model Results

| Fixed Effect Omnibus tests | | | | | | | |
| --- | --- | --- | --- | --- | --- | --- | --- |
|  | | X² | | df | | p | |
| More readable is human |  | 19.46880 |  | 1.00 |  | < .001 |  |
| Confidence |  | 2.95358 |  | 1.00 |  | 0.086 |  |
| Demographics: age |  | 0.13244 |  | 1.00 |  | 0.716 |  |
| Demographics: education\_ordinal |  | 0.29586 |  | 1.00 |  | 0.586 |  |
| Demographics: ai\_usage\_ordinal |  | 0.69590 |  | 1.00 |  | 0.404 |  |
| Demographics: gender merged |  | 0.15981 |  | 1.00 |  | 0.689 |  |
| Demographics: field\_merged |  | 10.29144 |  | 4.00 |  | 0.036 |  |
| Reaction time log |  | 0.02321 |  | 1.00 |  | 0.879 |  |
| GLS Euclidean distance |  | 0.00343 |  | 1.00 |  | 0.953 |  |
| GLS1 difference |  | 0.59204 |  | 1.00 |  | 0.442 |  |
| GLS2 difference |  | 3.41109 |  | 1.00 |  | 0.065 |  |
| GLS3 difference |  | 2.16118 |  | 1.00 |  | 0.142 |  |
| GLS4 difference |  | 0.67797 |  | 1.00 |  | 0.410 |  |
| id |  | 16.08902 |  | 1.00 |  | < .001 |  |
| Demographics: reading\_disorder\_merged |  | 1.71524 |  | 1.00 |  | 0.190 |  |
| GLS8 difference |  | 0.17682 |  | 1.00 |  | 0.674 |  |
| GLS7 difference |  | 0.79916 |  | 1.00 |  | 0.371 |  |
| GLS6 difference |  | 4.06814 |  | 1.00 |  | 0.044 |  |
| GLS5 difference |  | 0.22886 |  | 1.00 |  | 0.632 |  |
| Demographics: ai\_improve |  | 1.57010 |  | 1.00 |  | 0.210 |  |
| Demographics: ai\_risk |  | 0.03050 |  | 1.00 |  | 0.861 |  |
| Demographics: ai\_quality |  | 2.11651 |  | 1.00 |  | 0.146 |  |
| Demographics: ai\_lacks\_creativity |  | 0.45344 |  | 1.00 |  | 0.501 |  |
| Demographics: ai\_lacks\_understanding |  | 1.15976 |  | 1.00 |  | 0.282 |  |
| Demographics: ai\_grammar |  | 0.06892 |  | 1.00 |  | 0.793 |  |
| Demographics: ai\_avoidance |  | 5.27193 |  | 1.00 |  | 0.022 |  |
| Demographics: ai\_dumbing |  | 0.04171 |  | 1.00 |  | 0.838 |  |
| Demographics: ai\_fascination |  | 0.03826 |  | 1.00 |  | 0.845 |  |
|  |  |  |  |  |  |  |  |
| --- | --- | --- | --- | --- | --- | --- | --- |
|  | | | | | | | |
|  | | | | | | | |

| Fixed Effects Parameter Estimates | | | | | | | | | | | | | | | | | | | | | |
| --- | --- | --- | --- | --- | --- | --- | --- | --- | --- | --- | --- | --- | --- | --- | --- | --- | --- | --- | --- | --- | --- |
|  | | | | | | | | 95% Confidence Interval | | | |  | | 95% Exp(B) Confidence Interval | | | |  | | | |
| Names | | Effect | | Estimate | | SE | | Lower | | Upper | | exp(B) | | Lower | | Upper | | z | | p | |
| (Intercept) |  | (Intercept) |  | 1.75347 |  | 0.3499 |  | 1.06769 |  | 2.4393 |  | 5.775 |  | 2.9087 |  | 11.464 |  | 5.0114 |  | < .001 |  |
| More readable is human1 |  | 1 - 0 |  | 1.22118 |  | 0.2768 |  | 0.67873 |  | 1.7636 |  | 3.391 |  | 1.9714 |  | 5.834 |  | 4.4123 |  | < .001 |  |
| Confidence |  | Confidence |  | 0.08892 |  | 0.0517 |  | -0.01249 |  | 0.1903 |  | 1.093 |  | 0.9876 |  | 1.210 |  | 1.7186 |  | 0.086 |  |
| Demographics: age |  | Demographics: age |  | 0.00393 |  | 0.0108 |  | -0.01723 |  | 0.0251 |  | 1.004 |  | 0.9829 |  | 1.025 |  | 0.3639 |  | 0.716 |  |
| Demographics: education\_ordinal |  | Demographics: education\_ordinal |  | -0.07256 |  | 0.1334 |  | -0.33404 |  | 0.1889 |  | 0.930 |  | 0.7160 |  | 1.208 |  | -0.5439 |  | 0.586 |  |
| Demographics: ai\_usage\_ordinal |  | Demographics: ai\_usage\_ordinal |  | 0.05217 |  | 0.0625 |  | -0.07040 |  | 0.1747 |  | 1.054 |  | 0.9320 |  | 1.191 |  | 0.8342 |  | 0.404 |  |
| Demographics: gender merged1 |  | male - female |  | -0.08266 |  | 0.2068 |  | -0.48794 |  | 0.3226 |  | 0.921 |  | 0.6139 |  | 1.381 |  | -0.3998 |  | 0.689 |  |
| Demographics: field\_merged1 |  | human - cs |  | -1.70755 |  | 0.8003 |  | -3.27602 |  | -0.1391 |  | 0.181 |  | 0.0378 |  | 0.870 |  | -2.1338 |  | 0.033 |  |
| Demographics: field\_merged2 |  | nature - cs |  | -1.68028 |  | 0.8012 |  | -3.25057 |  | -0.1100 |  | 0.186 |  | 0.0388 |  | 0.896 |  | -2.0972 |  | 0.036 |  |
| Demographics: field\_merged3 |  | other - cs |  | -1.27117 |  | 0.8850 |  | -3.00583 |  | 0.4635 |  | 0.281 |  | 0.0495 |  | 1.590 |  | -1.4363 |  | 0.151 |  |
| Demographics: field\_merged4 |  | philology - cs |  | -1.17388 |  | 0.7908 |  | -2.72376 |  | 0.3760 |  | 0.309 |  | 0.0656 |  | 1.456 |  | -1.4845 |  | 0.138 |  |
| Reaction time log |  | Reaction time log |  | 0.01424 |  | 0.0935 |  | -0.16893 |  | 0.1974 |  | 1.014 |  | 0.8446 |  | 1.218 |  | 0.1523 |  | 0.879 |  |
| GLS Euclidean distance |  | GLS Euclidean distance |  | -0.00341 |  | 0.0581 |  | -0.11732 |  | 0.1105 |  | 0.997 |  | 0.8893 |  | 1.117 |  | -0.0586 |  | 0.953 |  |
| GLS1 difference |  | GLS1 difference |  | -0.08284 |  | 0.1077 |  | -0.29387 |  | 0.1282 |  | 0.920 |  | 0.7454 |  | 1.137 |  | -0.7694 |  | 0.442 |  |
| GLS2 difference |  | GLS2 difference |  | -0.28056 |  | 0.1519 |  | -0.57830 |  | 0.0172 |  | 0.755 |  | 0.5608 |  | 1.017 |  | -1.8469 |  | 0.065 |  |
| GLS3 difference |  | GLS3 difference |  | 0.06608 |  | 0.0449 |  | -0.02202 |  | 0.1542 |  | 1.068 |  | 0.9782 |  | 1.167 |  | 1.4701 |  | 0.142 |  |
| GLS4 difference |  | GLS4 difference |  | 0.04319 |  | 0.0525 |  | -0.05962 |  | 0.1460 |  | 1.044 |  | 0.9421 |  | 1.157 |  | 0.8234 |  | 0.410 |  |
| id |  | id |  | 0.05304 |  | 0.0132 |  | 0.02712 |  | 0.0790 |  | 1.054 |  | 1.0275 |  | 1.082 |  | 4.0111 |  | < .001 |  |
| Demographics: reading\_disorder\_merged1 |  | true - false |  | 0.72202 |  | 0.5513 |  | -0.35851 |  | 1.8025 |  | 2.059 |  | 0.6987 |  | 6.065 |  | 1.3097 |  | 0.190 |  |
| GLS8 difference |  | GLS8 difference |  | 0.01752 |  | 0.0417 |  | -0.06414 |  | 0.0992 |  | 1.018 |  | 0.9379 |  | 1.104 |  | 0.4205 |  | 0.674 |  |
| GLS7 difference |  | GLS7 difference |  | -0.04552 |  | 0.0509 |  | -0.14532 |  | 0.0543 |  | 0.956 |  | 0.8647 |  | 1.056 |  | -0.8940 |  | 0.371 |  |
| GLS6 difference |  | GLS6 difference |  | 0.07922 |  | 0.0393 |  | 0.00224 |  | 0.1562 |  | 1.082 |  | 1.0022 |  | 1.169 |  | 2.0170 |  | 0.044 |  |
| GLS5 difference |  | GLS5 difference |  | -0.03025 |  | 0.0632 |  | -0.15419 |  | 0.0937 |  | 0.970 |  | 0.8571 |  | 1.098 |  | -0.4784 |  | 0.632 |  |
| Demographics: ai\_improve |  | Demographics: ai\_improve |  | 0.09082 |  | 0.0725 |  | -0.05124 |  | 0.2329 |  | 1.095 |  | 0.9501 |  | 1.262 |  | 1.2530 |  | 0.210 |  |
| Demographics: ai\_risk |  | Demographics: ai\_risk |  | 0.01045 |  | 0.0598 |  | -0.10683 |  | 0.1277 |  | 1.011 |  | 0.8987 |  | 1.136 |  | 0.1746 |  | 0.861 |  |
| Demographics: ai\_quality |  | Demographics: ai\_quality |  | 0.09781 |  | 0.0672 |  | -0.03396 |  | 0.2296 |  | 1.103 |  | 0.9666 |  | 1.258 |  | 1.4548 |  | 0.146 |  |
| Demographics: ai\_lacks\_creativity |  | Demographics: ai\_lacks\_creativity |  | 0.04339 |  | 0.0644 |  | -0.08290 |  | 0.1697 |  | 1.044 |  | 0.9204 |  | 1.185 |  | 0.6734 |  | 0.501 |  |
| Demographics: ai\_lacks\_understanding |  | Demographics: ai\_lacks\_understanding |  | 0.06126 |  | 0.0569 |  | -0.05023 |  | 0.1728 |  | 1.063 |  | 0.9510 |  | 1.189 |  | 1.0769 |  | 0.282 |  |
| Demographics: ai\_grammar |  | Demographics: ai\_grammar |  | -0.01654 |  | 0.0630 |  | -0.14001 |  | 0.1069 |  | 0.984 |  | 0.8693 |  | 1.113 |  | -0.2625 |  | 0.793 |  |
| Demographics: ai\_avoidance |  | Demographics: ai\_avoidance |  | 0.12247 |  | 0.0533 |  | 0.01793 |  | 0.2270 |  | 1.130 |  | 1.0181 |  | 1.255 |  | 2.2961 |  | 0.022 |  |
| Demographics: ai\_dumbing |  | Demographics: ai\_dumbing |  | 0.01103 |  | 0.0540 |  | -0.09478 |  | 0.1168 |  | 1.011 |  | 0.9096 |  | 1.124 |  | 0.2042 |  | 0.838 |  |
| Demographics: ai\_fascination |  | Demographics: ai\_fascination |  | 0.01445 |  | 0.0739 |  | -0.13032 |  | 0.1592 |  | 1.015 |  | 0.8778 |  | 1.173 |  | 0.1956 |  | 0.845 |  |
|  |  |  |  |  |  |  |  |  |  |  |  |  |  |  |  |  |  |  |  |  |  |
| --- | --- | --- | --- | --- | --- | --- | --- | --- | --- | --- | --- | --- | --- | --- | --- | --- | --- | --- | --- | --- | --- |
|  | | | | | | | | | | | | | | | | | | | | | |
|  | | | | | | | | | | | | | | | | | | | | | |

| Random Components | | | | | | | | | |
| --- | --- | --- | --- | --- | --- | --- | --- | --- | --- |
| Groups | | Name | | SD | | Variance | | ICC | |
| Participant ID |  | More readable is human0 |  | 0.8707 |  | 0.75812 |  |  |  |
|  |  | More readable is human1 |  | 1.6450 |  | 2.70616 |  |  |  |
|  |  | Confidence |  | 0.2783 |  | 0.07746 |  |  |  |
|  |  | Reaction time log |  | 0.2246 |  | 0.05044 |  |  |  |
|  |  | GLS Euclidean distance |  | 0.2031 |  | 0.04125 |  |  |  |
|  |  | GLS1 difference |  | 0.6851 |  | 0.46940 |  |  |  |
|  |  | GLS2 difference |  | 0.6633 |  | 0.43995 |  |  |  |
|  |  | GLS3 difference |  | 0.1631 |  | 0.02660 |  |  |  |
|  |  | GLS4 difference |  | 0.0705 |  | 0.00497 |  |  |  |
|  |  | id |  | 0.0590 |  | 0.00348 |  |  |  |
|  |  | GLS8 difference |  | 0.1607 |  | 0.02584 |  |  |  |
|  |  | GLS7 difference |  | 0.1277 |  | 0.01631 |  |  |  |
|  |  | GLS6 difference |  | 0.1719 |  | 0.02954 |  |  |  |
|  |  | GLS5 difference |  | 0.2500 |  | 0.06249 |  |  |  |
| Residuals |  |  |  | 1.0000 |  | 1.00000 |  | . |  |
|  |  |  |  |  |  |  |  |  |  |
| --- | --- | --- | --- | --- | --- | --- | --- | --- | --- |
| Note. Number of Obs: 2074 , groups: Participant ID 122 | | | | | | | | | |
|  | | | | | | | | | |
|  | | | | | | | | | |

| Random Parameters correlations | | | | | | | |
| --- | --- | --- | --- | --- | --- | --- | --- |
| Groups | | Param.1 | | Param.2 | | Corr. | |
| Participant ID |  | More readable is human0 |  | More readable is human1 |  | -0.74893 |  |
|  |  | More readable is human0 |  | Confidence |  | 0.69299 |  |
|  |  | More readable is human0 |  | Reaction time log |  | 0.14039 |  |
|  |  | More readable is human0 |  | GLS Euclidean distance |  | -0.11741 |  |
|  |  | More readable is human0 |  | GLS1 difference |  | 0.00524 |  |
|  |  | More readable is human0 |  | GLS2 difference |  | -0.05658 |  |
|  |  | More readable is human0 |  | GLS3 difference |  | 0.16093 |  |
|  |  | More readable is human0 |  | GLS4 difference |  | 0.14308 |  |
|  |  | More readable is human0 |  | id |  | 0.39597 |  |
|  |  | More readable is human0 |  | GLS8 difference |  | -0.05064 |  |
|  |  | More readable is human0 |  | GLS7 difference |  | 0.10114 |  |
|  |  | More readable is human0 |  | GLS6 difference |  | 0.32191 |  |
|  |  | More readable is human0 |  | GLS5 difference |  | 0.30439 |  |
|  |  | More readable is human1 |  | Confidence |  | -0.36201 |  |
|  |  | More readable is human1 |  | Reaction time log |  | -0.10605 |  |
|  |  | More readable is human1 |  | GLS Euclidean distance |  | 0.18802 |  |
|  |  | More readable is human1 |  | GLS1 difference |  | 0.07698 |  |
|  |  | More readable is human1 |  | GLS2 difference |  | -0.43645 |  |
|  |  | More readable is human1 |  | GLS3 difference |  | -0.04425 |  |
|  |  | More readable is human1 |  | GLS4 difference |  | 0.28439 |  |
|  |  | More readable is human1 |  | id |  | -0.20016 |  |
|  |  | More readable is human1 |  | GLS8 difference |  | 0.09590 |  |
|  |  | More readable is human1 |  | GLS7 difference |  | -0.08440 |  |
|  |  | More readable is human1 |  | GLS6 difference |  | -0.14409 |  |
|  |  | More readable is human1 |  | GLS5 difference |  | 0.22300 |  |
|  |  | Confidence |  | Reaction time log |  | 0.62084 |  |
|  |  | Confidence |  | GLS Euclidean distance |  | 5.82e-4 |  |
|  |  | Confidence |  | GLS1 difference |  | 0.13458 |  |
|  |  | Confidence |  | GLS2 difference |  | -0.32724 |  |
|  |  | Confidence |  | GLS3 difference |  | -0.16975 |  |
|  |  | Confidence |  | GLS4 difference |  | -0.02755 |  |
|  |  | Confidence |  | id |  | 0.70806 |  |
|  |  | Confidence |  | GLS8 difference |  | -0.56421 |  |
|  |  | Confidence |  | GLS7 difference |  | 0.27740 |  |
|  |  | Confidence |  | GLS6 difference |  | 0.70559 |  |
|  |  | Confidence |  | GLS5 difference |  | 0.43945 |  |
|  |  | Reaction time log |  | GLS Euclidean distance |  | -0.47991 |  |
|  |  | Reaction time log |  | GLS1 difference |  | 0.33138 |  |
|  |  | Reaction time log |  | GLS2 difference |  | -0.31651 |  |
|  |  | Reaction time log |  | GLS3 difference |  | -0.23088 |  |
|  |  | Reaction time log |  | GLS4 difference |  | -0.15295 |  |
|  |  | Reaction time log |  | id |  | 0.90143 |  |
|  |  | Reaction time log |  | GLS8 difference |  | -0.85447 |  |
|  |  | Reaction time log |  | GLS7 difference |  | 0.29589 |  |
|  |  | Reaction time log |  | GLS6 difference |  | 0.86667 |  |
|  |  | Reaction time log |  | GLS5 difference |  | 0.18886 |  |
|  |  | GLS Euclidean distance |  | GLS1 difference |  | -0.42465 |  |
|  |  | GLS Euclidean distance |  | GLS2 difference |  | 0.31367 |  |
|  |  | GLS Euclidean distance |  | GLS3 difference |  | -0.41959 |  |
|  |  | GLS Euclidean distance |  | GLS4 difference |  | -0.36307 |  |
|  |  | GLS Euclidean distance |  | id |  | -0.63713 |  |
|  |  | GLS Euclidean distance |  | GLS8 difference |  | 0.30621 |  |
|  |  | GLS Euclidean distance |  | GLS7 difference |  | 0.09862 |  |
|  |  | GLS Euclidean distance |  | GLS6 difference |  | -0.48188 |  |
|  |  | GLS Euclidean distance |  | GLS5 difference |  | 0.02764 |  |
|  |  | GLS1 difference |  | GLS2 difference |  | -0.57781 |  |
|  |  | GLS1 difference |  | GLS3 difference |  | 0.57626 |  |
|  |  | GLS1 difference |  | GLS4 difference |  | 0.46697 |  |
|  |  | GLS1 difference |  | id |  | 0.29781 |  |
|  |  | GLS1 difference |  | GLS8 difference |  | -0.64415 |  |
|  |  | GLS1 difference |  | GLS7 difference |  | 0.52507 |  |
|  |  | GLS1 difference |  | GLS6 difference |  | 0.66018 |  |
|  |  | GLS1 difference |  | GLS5 difference |  | 0.49995 |  |
|  |  | GLS2 difference |  | GLS3 difference |  | -0.57099 |  |
|  |  | GLS2 difference |  | GLS4 difference |  | -0.81916 |  |
|  |  | GLS2 difference |  | id |  | -0.47639 |  |
|  |  | GLS2 difference |  | GLS8 difference |  | 0.35691 |  |
|  |  | GLS2 difference |  | GLS7 difference |  | 0.09307 |  |
|  |  | GLS2 difference |  | GLS6 difference |  | -0.49190 |  |
|  |  | GLS2 difference |  | GLS5 difference |  | -0.48264 |  |
|  |  | GLS3 difference |  | GLS4 difference |  | 0.85285 |  |
|  |  | GLS3 difference |  | id |  | -0.00918 |  |
|  |  | GLS3 difference |  | GLS8 difference |  | 0.03398 |  |
|  |  | GLS3 difference |  | GLS7 difference |  | -0.26463 |  |
|  |  | GLS3 difference |  | GLS6 difference |  | 0.03420 |  |
|  |  | GLS3 difference |  | GLS5 difference |  | 0.04830 |  |
|  |  | GLS4 difference |  | id |  | 0.13398 |  |
|  |  | GLS4 difference |  | GLS8 difference |  | 0.10999 |  |
|  |  | GLS4 difference |  | GLS7 difference |  | -0.30214 |  |
|  |  | GLS4 difference |  | GLS6 difference |  | 0.09777 |  |
|  |  | GLS4 difference |  | GLS5 difference |  | 0.33781 |  |
|  |  | id |  | GLS8 difference |  | -0.67682 |  |
|  |  | id |  | GLS7 difference |  | 0.09342 |  |
|  |  | id |  | GLS6 difference |  | 0.84847 |  |
|  |  | id |  | GLS5 difference |  | 0.25491 |  |
|  |  | GLS8 difference |  | GLS7 difference |  | -0.51185 |  |
|  |  | GLS8 difference |  | GLS6 difference |  | -0.88612 |  |
|  |  | GLS8 difference |  | GLS5 difference |  | -0.25024 |  |
|  |  | GLS7 difference |  | GLS6 difference |  | 0.54724 |  |
|  |  | GLS7 difference |  | GLS5 difference |  | 0.66339 |  |
|  |  | GLS6 difference |  | GLS5 difference |  | 0.53784 |  |
|  |  |  |  |  |  |  |  |
| --- | --- | --- | --- | --- | --- | --- | --- |
|  | | | | | | | |
|  | | | | | | | |

## Post Hoc Tests

| Post Hoc Comparisons - More readable is human | | | | | | | | | | | | | |
| --- | --- | --- | --- | --- | --- | --- | --- | --- | --- | --- | --- | --- | --- |
| Comparison | | | | | |  | | | | | | | |
| More readable is human | |  | | More readable is human | | exp(B) | | SE | | z | | p_bonferroni_ | |
| 0 |  | - |  | 1 |  | 0.295 |  | 0.0816 |  | -4.41 |  | < .001 |  |
|  |  |  |  |  |  |  |  |  |  |  |  |  |  |
| --- | --- | --- | --- | --- | --- | --- | --- | --- | --- | --- | --- | --- | --- |
|  | | | | | | | | | | | | | |
|  | | | | | | | | | | | | | |

| Post Hoc Comparisons - Demographics: gender merged | | | | | | | | | | | | | |
| --- | --- | --- | --- | --- | --- | --- | --- | --- | --- | --- | --- | --- | --- |
| Comparison | | | | | |  | | | | | | | |
| Demographics: gender merged | |  | | Demographics: gender merged | | exp(B) | | SE | | z | | p_bonferroni_ | |
| female |  | - |  | male |  | 1.09 |  | 0.225 |  | 0.400 |  | 0.689 |  |
|  |  |  |  |  |  |  |  |  |  |  |  |  |  |
| --- | --- | --- | --- | --- | --- | --- | --- | --- | --- | --- | --- | --- | --- |
|  | | | | | | | | | | | | | |
|  | | | | | | | | | | | | | |

| Post Hoc Comparisons - Demographics: field\_merged | | | | | | | | | | | | | |
| --- | --- | --- | --- | --- | --- | --- | --- | --- | --- | --- | --- | --- | --- |
| Comparison | | | | | |  | | | | | | | |
| Demographics: field\_merged | |  | | Demographics: field\_merged | | exp(B) | | SE | | z | | p_bonferroni_ | |
| cs |  | - |  | human |  | 5.515 |  | 4.414 |  | 2.134 |  | 0.329 |  |
| cs |  | - |  | nature |  | 5.367 |  | 4.300 |  | 2.097 |  | 0.360 |  |
| cs |  | - |  | other |  | 3.565 |  | 3.155 |  | 1.436 |  | 1.000 |  |
| cs |  | - |  | philology |  | 3.235 |  | 2.558 |  | 1.484 |  | 1.000 |  |
| human |  | - |  | nature |  | 0.973 |  | 0.199 |  | -0.134 |  | 1.000 |  |
| human |  | - |  | other |  | 0.646 |  | 0.270 |  | -1.045 |  | 1.000 |  |
| human |  | - |  | philology |  | 0.586 |  | 0.129 |  | -2.433 |  | 0.150 |  |
| nature |  | - |  | other |  | 0.664 |  | 0.286 |  | -0.950 |  | 1.000 |  |
| nature |  | - |  | philology |  | 0.603 |  | 0.143 |  | -2.139 |  | 0.325 |  |
| other |  | - |  | philology |  | 0.907 |  | 0.394 |  | -0.224 |  | 1.000 |  |
|  |  |  |  |  |  |  |  |  |  |  |  |  |  |
| --- | --- | --- | --- | --- | --- | --- | --- | --- | --- | --- | --- | --- | --- |
|  | | | | | | | | | | | | | |
|  | | | | | | | | | | | | | |

| Post Hoc Comparisons - Demographics: reading\_disorder\_merged | | | | | | | | | | | | | |
| --- | --- | --- | --- | --- | --- | --- | --- | --- | --- | --- | --- | --- | --- |
| Comparison | | | | | |  | | | | | | | |
| Demographics: reading\_disorder\_merged | |  | | Demographics: reading\_disorder\_merged | | exp(B) | | SE | | z | | p_bonferroni_ | |
| false |  | - |  | true |  | 0.486 |  | 0.268 |  | -1.31 |  | 0.190 |  |
|  |  |  |  |  |  |  |  |  |  |  |  |  |  |
| --- | --- | --- | --- | --- | --- | --- | --- | --- | --- | --- | --- | --- | --- |
|  | | | | | | | | | | | | | |
|  | | | | | | | | | | | | | |

# References

[1]
The jamovi project (2022). *jamovi*. (Version 2.3) [Computer Software]. Retrieved from https://www.jamovi.org.

[2]
R Core Team (2021). *R: A Language and environment for statistical computing*. (Version 4.1) [Computer software]. Retrieved from https://cran.r-project.org. (R packages retrieved from MRAN snapshot 2022-01-01).

[3]
Gallucci, M. (2019). *GAMLj: General analyses for linear models*. [jamovi module]. Retrieved from https://gamlj.github.io/.
